# 3D‐Printed Sodium Alginate/Gelatin/Black Phosphorus Nanosheet Hydrogel Patches for Urethral Stricture Repair

**DOI:** 10.1155/ijbm/5745826

**Published:** 2026-10-04

**Authors:** Zhidong Ma, Jiafu Liu, Wenzhuo Fang, Jie Yan, Wang Song, Xi Yang, Wenyao Li, Kaile Zhang

**Affiliations:** ^1^ School of Materials Science and Engineering Shanghai University of Engineering Science Shanghai 201620 China sues.edu.cn; ^2^ Department of Urology Shanghai Sixth People’s Hospital Affiliated to Shanghai Jiao Tong University School of Medicine Shanghai 200233 China sjtu.edu.cn; ^3^ Novaprint Therapeutics Suzhou Co., Ltd Suzhou 215000 China

**Keywords:** 3D printing, black phosphorus nanosheets, hydrogel patch, urethral stricture

## Abstract

The urethral stricture is still a clinical challenge, where bioactive scaffold materials capable of providing critical biological hints are needed to help regenerate the function and also reduce excessive fibrosis. Black phosphorus nanosheets (BPNSs) have been identified as promising nanomaterials in tissue engineering; however, their effects on urethral repair, depending on their concentration, remain poorly understood. In this study, we fabricated a porous sodium alginate/gelatin/black phosphorus nanosheet (SA/Gel/BPNS) composite hydrogel patch based on extrusion‐based 3D printing technology. The physicochemical properties and biocompatibility of the scaffolds were characterized. The biological effects of varying BPNS concentrations on human foreskin fibroblast (HFF) behaviors were systematically investigated in vitro. Furthermore, the regenerative potential of the patches was evaluated in a rabbit model of urethral injury through macroscopic and histological assessments. In vitro assays revealed that BPNS exerts a distinct biphasic regulatory effect on HFFs: Low concentrations (0.25 μg·mL^−1^) significantly promoted cell migration and the expression of pro‐repair markers (fibronectin, COL1, and *α*‐SMA), while high concentrations (4 μg·mL^−1^) inhibited fibrotic marker expression. Transcriptomic analysis further indicated that low‐concentration BPNS was associated with the PI3K‐Akt signaling pathway, whereas high‐concentration BPNS mainly affected ECM–receptor interaction, focal adhesion, and Rap1 signaling pathways. In vivo results demonstrated that the SA/Gel/BPNS_0.25_ group had the best repair performance, characterized by an intact, smooth urethral surface, enhanced epithelial remodeling, and organized tissue regeneration with reduced fibrous scar deposition compared to the control groups. The SA/Gel/BPNS patch integrates mechanical scaffolding with concentration‐dependent biomodulation. Through optimization of BPNS concentrations, this patch effectively facilitates functional urethral healing and mitigates secondary scarring, offering a potential bioengineering solution for the clinical treatment of urethral strictures.

## 1. Introduction

Urethral stricture is one of the most common urological conditions characterized by luminal narrowing due to fibrosis, typically secondary to trauma, infection, iatrogenic injury, or inflammatory disease [1, 2]. The progressive nature of fibrosis and subsequent scarring impairs normal voiding function, significantly diminishing patient quality of life [3]. Despite substantial scientific advancements, evidence suggests that clinical understanding of urethral stricture surgery remains insufficient and that patients often face a paucity of effective therapeutic options [4–6]. Current clinical management primarily relies on urethral dilation, direct vision internal urethrotomy, and urethroplasty. Compared with urethral dilation and urethrotomy, urethroplasty is considered the most effective gold standard method [7, 8]. Nevertheless, its recurrence rate remains relatively high, particularly for long‐segment or complex urethral strictures. Surgical reconstruction involves appropriate mucosal substitutes, with autologous grafts (penile skin flaps or buccal mucosa) being the most commonly used [9]. However, the clinical utility of autologous materials is constrained by donor‐site morbidity, limited tissue availability, and potential complications at the harvest site. Therefore, there is a critical clinical imperative to develop alternative tissue engineering strategies for urethral repair [10–12].

Recent years have seen tissue engineering emerge as a promising frontier for urethral regeneration. Yet, despite progress in foundational research, translating these findings into clinical practice remains challenging [13–15]. Various biomaterials have been explored for the fabrication of urethral repair scaffolds, including acellular matrices and natural polymers, such as collagen, chitosan, and sodium alginate (SA) [16–18]. Among these, hydrogels have gained significant attention due to their high‐water content, excellent biocompatibility, and ability to mimic the microenvironmental characteristics of the native extracellular matrix (ECM). Nevertheless, conventional hydrogel scaffolds often suffer from inadequate mechanical strength, limited antimicrobial properties, and a diminished capacity to modulate key biological processes, such as angiogenesis and tissue remodeling [19, 20]. Besides, traditional fabrication methods lack precise control over scaffold architecture, which is essential for guiding cellular infiltration and facilitating nutrient exchange. Therefore, developing hydrogel systems that integrate mechanical adaptability, biofunctionality, and structural controllability is essential to advance their application in urethral repair.

Natural polymer‐based hydrogels have become pivotal scaffold materials in regenerative medicine due to their biomimetic ECM properties [21]. SA, a linear polysaccharide, is widely utilized for its rapid and mild ionic crosslinking mechanism—frequently described by the “egg‐box model”—which ensures high cell viability during encapsulation [21, 22]. However, pristine SA exhibits inherent limitations, notably a lack of cell‐binding sites on its backbone, leading to poor cellular response [23–25]. To address this, incorporating gelatin (Gel), a collagen derivative rich in Arg‐Gly‐Asp (RGD) sequences, has proven to be an effective strategy for enhancing the bioplasticity of the inert SA framework [26–28]. While Gel provides critical cell‐recognition motifs, its mechanical instability at physiological temperatures necessitates reinforcement. Thus, the strategic hybridization of SA and Gel creates a synergistic platform: SA provides a robust structural template via ionic coordination, while Gel serves as a biofunctional modulator. Together, they allow for the tailoring of physicochemical properties to meet the complex requirements of tissue regeneration.

Although SA/Gel composite hydrogels are ideal in simulating the ECM, pure polymeric networks are unable to provide a high mechanical strength alongside multifunctional bioinductivity. As a result, incorporating functional inorganic nanofillers has emerged as a crucial strategy for enhancing scaffold performance. Recently, black phosphorus nanosheets (BPNS), an emerging two‐dimensional (2D) bioactive material, have garnered significant attention in regenerative medicine owing to their unique physicochemical properties. Distinct from conventional carbon‐based nanomaterials, BPNSs exhibit excellent biodegradability; they undergo gradual oxidative degradation into nontoxic phosphate ions under physiological conditions. These degradation products not only integrate into endogenous phosphorus metabolism but also serve as biochemical signaling molecules to facilitate tissue repair [29–31]. According to earlier research, BPNSs have been shown to significantly enhance the recruitment of endothelial cells and neovascularization by inducing particular signaling pathways [32, 33]. Moreover, due to their considerable negative surface charge and their ability to produce singlet oxygen, BPNSs demonstrate a wide‐spectrum antimicrobial effect, which is an effective measure against postimplantation infection. At the same time, the high anisotropy of BPNSs enables strong interfacial interactions with SA/Gel polymeric chains via physical entanglement and hydrogen bonding, thereby substantially augmenting the mechanical stiffness and structural stability of the composite hydrogel [34]. Therefore, integrating BPNSs into the SA/Gel matrix presents a promising approach to constructing a multidimensional scaffold system that synergistically combines robust mechanical support, highly efficient antimicrobial properties, and active bioinductivity.

In addition to the inherent physicochemical and biological characteristics of biomaterials, the spatial organization of scaffold is also important in directing tissue regeneration [35]. Traditional hydrogel fabrication techniques can be difficult to use to create customizable macroscopic geometries and exact control over microscopic pore structure. Three‐dimensional (3D) printing, an advanced additive manufacturing technology, enables the fabrication of scaffolds with high structural fidelity while allowing precise regulation of both external geometry and internal pore architecture. This capability makes it possible to construct highly interconnected porous networks with well‐defined structures and customizable shapes [36, 37]. The structural controllability at both macro‐ and microscale levels is essential for promoting cell infiltration, facilitating vascularization, and ensuring efficient exchange of nutrients and metabolic waste, thereby helping to mitigate tissue necrosis during the regenerative process [33, 35]. Thus, integrating multifunctional nanocomposite systems—such as BPNSs‐incorporated SA/Gel hydrogels—into printable inks and combining them with 3D printing technology represent a promising strategy for urethral tissue engineering. This approach not only enables precise adaptation to the complex anatomical geometry of urethral defects but also facilitates the fabrication of biomimetic scaffolds with tailored architectures and enhanced biological functionality, ultimately promoting functional urethral regeneration.

This study developed a 3D‐printed SA/Gel/BPNS composite hydrogel patch for urethral stricture repair. Its physicochemical properties were systematically characterized, followed by in vitro evaluation of its cytocompatibility and the concentration‐dependent effects of BPNS on fibroblast proliferation, migration, and fibrosis‐related responses. The reparative effects of the hydrogel patch were further evaluated in a rabbit urethral defect model. By comparing different BPNS concentrations, this study aimed to explore the relationship between BPNS concentration, fibroblast responses, and tissue remodeling, providing a potential biomaterial platform for urethral tissue repair (Scheme 1).

**SCHEME 1:**
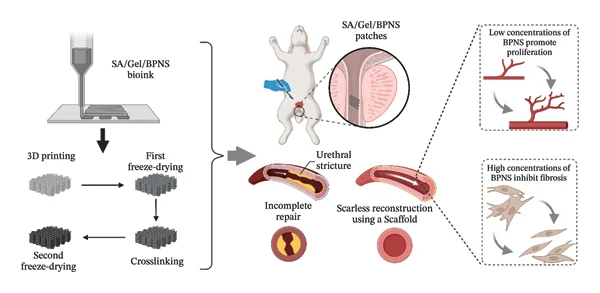
The schematic displays the fabrication and therapeutic mechanism of the composite hydrogel patch. A porous SA/Gel/BPNS hydrogel patch is fabricated via extrusion‐based 3D printing technology. In vitro investigation of the concentration‐dependent biphasic regulatory effects of BPNS on HFFs. Low concentrations (BP_0.25_) promote cell migration and pro‐repair activities, while high concentrations (BP_4_) inhibit excessive fibrotic responses. In vivo evaluation in a rabbit model of urethral injury. The SA/Gel/BP patch effectively facilitates functional urethral healing, characterized by enhanced epithelial remodeling, organized tissue regeneration, and minimized scar formation, offering a promising bioengineering strategy for clinical urethral stricture management. Created in BioRender. Ma, Z. (2026) https://BioRender.com/kd6ost3.

## 2. Materials and Methods

### 2.1. Preparation of BPNS‐Loaded Hydrogels

BPNS (purity > 99.998%, XFNANO, Nanjing, China) dispersions were prepared at an initial concentration of 1 mg·mL^−1^ by dispersing 20 mg of BPNS in 20 mL of double‐distilled water (*dd*H_2_O), followed by ultrasonication at 40 KHz for 1 h. SA (2% w/v, Sigma‐Aldrich) and Gel (2% w/v, Type A, porcine skin, ∼300 Bloom, Sigma‐Aldrich) were dissolved in *dd*H_2_O. The BPNS group served as the control. Solutions containing 0, 0.25, 1, 4, 16, and 64 μg·mL^−1^ of BPNS were added to SA/Gel solutions. The resulting sodium alginate/gelatin/black‐scaled nanosheet composite hydrogel solutions were designated as SA/Gel, SA/Gel/BPNS_0.25_, SA/Gel/BPNS_1_, SA/Gel/BPNS_4_, SA/Gel/BPNS_16_, and SA/Gel/BPNS_64_, respectively. The detailed preparation parameters for each formulation, including the BPNS stock concentration, volume added, and final precursor concentration, are summarized in Table S1. To maintain constant concentrations of SA and Gel, sterilized water and BPNS dispersions were mixed in specific ratios. For example, to prepare 20 mL of the SA/Gel/BPNS_0.25_ precursor, 5 μL of BPNS stock dispersion (1 mg·mL^−1^) was added to the aqueous phase, followed by the addition of 400 mg SA and 400 mg Gel. The mixture was stirred at 37°C for 24 h until completely dissolved and then allowed to stand for degassing before use.

### 2.2. 3D Printing

In this study, the SA/Gel/BPNS scaffold was printed using the Organ Printing United System (OPUS, Novaprint Therapeutics Suzhou Co., Ltd), which incorporates multinozzle printing and multimaterial mixing. The scaffold model was designed as a grid structure (15 × 15 × 0.75 mm^3^) and converted into G‐code via slicing software, featuring a 5‐layer stack with a 1.5 mm pore size. A 200 μm inner diameter nozzle was utilized. Given the high fluidity of the hydrogel at 37°C, the loaded syringe was precooled at 4°C for 8–10 min to achieve printable viscosity. The printing chamber was maintained at 19°C. The SA/Gel/BPNS scaffold was deposited layer by layer on the bioprinter platform. After printing each scaffold, it was placed in a −80°C ultra‐low temperature freezer. Subsequently, it underwent freeze‐drying in a vacuum freeze dryer for 12 h. Next, each freeze‐dried scaffold (15 × 15 × 0.75 mm^3^) was completely immersed in 5 mL of 5% CaCl_2_ solution (MW 110.98 g·mol^−1^, Sigma‐Aldrich) and crosslinked for 30 min. The scaffold was then thoroughly rinsed with sterile water to remove residual CaCl_2_, followed by secondary freeze‐drying. Subsequently, the Ca^2+^‐crosslinked scaffold was immersed in 5 mL of an EDC/NHS solution containing 50 mM EDC and 10 mM NHS and incubated at room temperature for 2 h to promote covalent coupling between accessible carboxyl groups in the alginate network and primary amino groups of gelatin. After the reaction, the scaffold was thoroughly rinsed with sterile water to remove residual EDC, NHS, and reaction by‐products, followed by secondary freeze‐drying. The resulting scaffold was stored in a vacuum container until further use.

### 2.3. Fourier Transform Infrared Spectroscopy (FTIR)

Functional groups of the SA/Gel/BPNS composite hydrogel were characterized using FTIR. The prepared hydrogel was freeze‐dried in a vacuum freeze dryer, finely ground with KBr (1:100), and compressed into a thin film for infrared spectroscopy measurements. The measurement range was 500–4000 cm^−1^.

### 2.4. Raman Spectroscopy

Raman spectroscopy was employed to qualitatively characterize BPNS in the composite hydrogel. Freeze‐dried SA/Gel and SA/Gel/BPNS samples were placed on clean microscope slides and analyzed using a Raman spectrometer with a 785 nm excitation laser. Raman spectra were collected over the range of 300–500 cm^−1^, covering the characteristic vibrational bands of BPNS.

### 2.5. Scanning Electron Microscopy (SEM)

The morphology of the SA/Gel/BPNS composite hydrogel was observed using SEM. In brief, samples were prepared in 24‐well plates (0.4 cm height). They were first frozen at 4°C and −80°C for 2 h, respectively, then freeze‐dried overnight in a vacuum freeze dryer. Subsequently, the freeze‐dried hydrogel samples were crosslinked with 5% CaCl_2_ for 30 min and thoroughly washed with sterile water to remove excess crosslinking agent. They were freeze‐dried again and stored in vacuum containers. The dried scaffolds were cut to size, placed on a stage coated with conductive adhesive, and finally gold‐sputtered in a vacuum sputter coater. Surface morphologies of different scaffold materials were observed under SEM at an acceleration voltage of 15 kV. Pore size and porosity distributions of the hydrogels were calculated using ImageJ visualization software.

### 2.6. Swelling and Biodegradation

The swelling behavior of SA/Gel/BPNS hydrogels containing different BPNS concentrations (0, 0.25, 1, 4, 16, and 64 μg·mL^−1^) was evaluated using the gravimetric method. Freeze‐dried hydrogel samples were immersed in PBS at room temperature for 5 days. After immersion, the samples were removed, and excess surface liquid was gently removed using filter paper before weighing. Three independent samples were tested for each formulation.

(1) SR%=ww−wdwd∗100%,

where *w*_*d*_ is the weight of the freeze‐dried hydrogel sample, and *w*_*w*_ is the weight of the hydrogel after water absorption.

The in vitro degradation performance of SA/Gel hydrogels at different BPNS concentrations was quantitatively evaluated. These samples were immersed in sterile water for 7, 14, 21, and 30 days before removal. They were then freeze‐dried, and the freeze‐dried samples were weighed. *W*_*I*_ denotes the initial dry mass, and *W*_*T*_ denotes the mass after immersion for *T* days (*T* = 7, 14, 21, and 30 days). Finally, the residual weight is calculated using the following formula. These experiments were conducted in triplicate.

(2) Mass remaining%=WTW1∗100%.

### 2.7. Cell Culture and Biocompatibility

Human foreskin fibroblasts (HFFs) were isolated from collected human foreskin tissue. Fibroblasts from the third to tenth passages were cultured in T‐75 flasks containing high‐glucose DMEM (Gibco) medium supplemented with 10% fetal bovine serum (FBS, Gibco) and 1% penicillin–streptomycin (Gibco), maintained at 37°C in a 5% CO_2_ incubator. Cell status was monitored under a microscope, and passages were performed when cells reached 80%–90% confluence. First, the culture medium was aspirated and washed with PBS (pH 7.4; Servicebio). Cells were then digested with 0.25% trypsin–EDTA (Gibco) for 1.5–2 min. Add complete medium (3–4 times the volume of trypsin) to terminate digestion. Finally, gently resuspend cells by pipetting and collect them in a sterile centrifuge tube. Centrifuge at 1000 rpm for 5 min, remove the supernatant, and resuspend the cell pellet in complete medium to obtain a cell suspension.

### 2.8. Effects of BPNS on Cell Migration

HFFs were seeded at a density of 2 × 10^5^ cells/well in 24‐well plates. When the cell confluence reached 80%–90%, a uniform scratch was created using a 1000 μL pipette tip. The cells were gently washed with PBS to remove detached cells, and the initial scratch images were captured using an inverted phase‐contrast microscope (*W*_0_). BPNS dispersions at the indicated concentrations were then prepared in DMEM containing 1% FBS and added to the corresponding wells. The low‐serum condition was used to minimize the contribution of cell proliferation while maintaining cell viability during the assay. After 24 h of culture, the same scratch areas were imaged again, and the wound width was recorded as Wt. Wound closure is calculated using the following equation. The experiment was repeated three times.

(3) Cell migration rate%=Wd0−WdtWd0∗100%.

### 2.9. Cytotoxicity

To qualitatively assess the biocompatibility of the nanocomposite hydrogels, a Calcein‐AM/PI Double Staining Kit was employed. Briefly, Calcein‐AM penetrates the cell membrane of viable cells and is hydrolyzed by intracellular esterases to produce green fluorescence (490 nm). Conversely, propidium iodide (PI) enters cells with compromised membranes, intercalating into the DNA to emit red fluorescence (535 nm), thereby identifying dead cells. Cells were seeded onto the hydrogel surfaces at a density of 5 × 10^4^ cells·mL^−1^. The staining solution, containing 2 μM Calcein‐AM and 4 μM PI, was prepared in sterile PBS and protected from light using aluminum foil. After 30 min of incubation in the dark, the samples were rinsed three times with PBS to remove excess dye. Images were captured using a fluorescence microscope equipped with an advanced imaging system. Live and dead cells were identified by green and red fluorescence, respectively.

The cytotoxicity of BPNS toward fibroblasts and the subsequent cell proliferation were quantitatively evaluated using a Cell Counting Kit‐8 (CCK‐8). The assay is based on the reduction of WST‐8 [2‐(2‐methoxy‐4‐nitrophenyl)‐3‐(4‐nitrophenyl)‐5‐(2,4‐disulfophenyl)‐2H‐tetrazolium, monosodium salt] by intracellular dehydrogenases to a water‐soluble, orange‐colored formazan product. The amount of formazan generated is directly proportional to the number of viable cells. Briefly, HFFs were seeded onto the SA/Gel/BPNS composite hydrogels (or SA/Gel control groups) and cultured for 1, 3, and 5 days. At each time point, the samples were rinsed three times with PBS. Subsequently, a CCK‐8 working solution (containing 10% CCK‐8 reagent in complete culture medium) was added to each well. After 4 h of incubation in a CO_2_ incubator (37°C, protected from light), 200 μL of the supernatant from each well was transferred to a 96‐well plate. Wells containing only the CCK‐8 working solution served as the blank control. The optical density (OD) was measured at 450 nm using a microplate reader. Each group was tested in quintuplicate (*n* = 5).

### 2.10. Western Blot (WB)

WB is commonly used to identify a certain protein and can be used for qualitative and semiquantitative protein analyses. At the point when the HFFs had adhered to the walls of the plate wells, the number of cells was approximately 1 × 10^6^–1 × 10^7^. The cells were washed 2–3 times with precooled PBS, and then 200 μL of strong RIPA lysis buffer (Beyotime Biotechnology, Shanghai, China) containing PMSF was added. The cells were scraped, and the lysate was centrifuged and stored in a centrifuge tube. According to the operating instructions of the BCA protein quantification kit (Beyotime Biotechnology, Shanghai, China), the absorbance values of the different experimental groups were measured at a wavelength of 562 nm. The protein concentrations of the samples were determined using a standard curve. SDS‐PAGE was performed on a 10% gel, and after electrophoresis, membrane transfer, blocking, and antibody hybridization were performed. TBST was used to wash the membrane three times, and the membrane was scanned with an imaging system. Protein detection was performed by colorimetric and luminescent measurements of grayscale values to determine the expression of differentially expressed proteins. The proteins to be detected included fibronectin, collagen I, and *α*‐SMA, with *β*‐actin serving as the internal reference.

### 2.11. Reverse Transcription Polymerase Chain Reaction (RT‐PCR)

Total RNA was extracted using TRIzol reagent (Invitrogen, USA) according to the manufacturer’s instructions. RNA purity and concentration were assessed spectrophotometrically. One microgram of total RNA was reverse‐transcribed into complementary DNA (cDNA) using a commercial cDNA synthesis kit according to the manufacturer’s protocol. Quantitative real‐time PCR (RT‐qPCR) was performed using SYBR Green Pro Taq HS qPCR Kit II (Rox Plus) (Accurate Biotechnology [Hunan] Co., Ltd., Changsha, China; AG11719) on a StepOnePlus Real‐Time PCR System (Applied Biosystems, USA). *β*‐Actin was used as the internal reference gene, and the relative expression levels of target genes were calculated using the 2−ΔΔCt method. The primer sequences used for RT‐qPCR are listed in Table S2.

### 2.12. RNA Sequencing and Bioinformatics Analysis

HFFs from the control, BPNS_0.25_, and BPNS_4_ groups were collected for RNA sequencing, with three independent biological replicates included in each group (nine samples in total). RNA library preparation and sequencing were performed by OE Biotech Co., Ltd. (Shanghai, China). Raw sequencing reads were quality‐filtered using fastp (v0.20.1), and sequencing quality was assessed using FastQC (v0.11.9) and RSeQC (v4.0.0). Clean reads were aligned to the human reference genome GRCh38.p13 using HISAT2 (v2.1.0). Gene‐level read counts were obtained using HTSeq‐count (v0.11.2), and gene expression levels were calculated as fragments per kilobase of transcript per million mapped reads (FPKM). Differential expression analysis was performed based on gene‐level counts using DESeq2 (v1.22.2). Genes with |log2 fold change| > 1 and *q*‐value < 0.05 were considered differentially expressed genes (DEGs). Gene Ontology (GO) enrichment analysis was performed for the DEGs using a hypergeometric test, with Fisher’s exact test used to evaluate enrichment significance in the biological process, cellular component, and molecular function categories. Kyoto Encyclopedia of Genes and Genomes (KEGG) pathway enrichment analysis was performed using the KEGG database, and enrichment significance was assessed using a hypergeometric test.

### 2.13. Urethral Injury Animal Model

To evaluate the in vivo biocompatibility and reparative effects of the 3D‐printed SA/Gel/BPNS scaffolds, the scaffolds were implanted into rabbit urethral defects. Adult male New Zealand White rabbits were selected as experimental animals because their urethral anatomy and tissue organization share certain similarities with those of humans. A total of 12 adult male New Zealand White rabbits, with an average body weight of approximately 2.5 kg, were included in the study and randomly allocated into four groups (*n* = 3 animals per group): control, SA/Gel/BPNS_0_, SA/Gel/BPNS_0.25_, and SA/Gel/BPNS_4_. The control group underwent urethral defect creation without scaffold implantation, while the SA/Gel/BPNS_0_, SA/Gel/BPNS_0.25_, and SA/Gel/BPNS_4_ groups received the corresponding hydrogel scaffolds containing 0, 0.25, and 4 μg·mL^−1^ BPNS, respectively.

The rabbit urethral defect model was established as follows. Before implantation, the 3D‐printed scaffolds (15 × 15 × 0.75 mm^3^) were aseptically trimmed into 15 × 5 × 0.75 mm^3^ patches and sterilized by ultraviolet (UV) irradiation for 45 min. Anesthesia was induced via the auricular vein using pentobarbital. After disinfection of the surgical area, an incision was made to expose the anterior urethra. A ventral urethral mucosal defect measuring approximately 2.0 × 0.8 cm was created. The prepared hydrogel patch (15 × 5 × 0.75 mm) was positioned over the defect and sutured to the defect margins using 6–0 absorbable polylactic acid sutures. The urethral defect was subsequently closed using 6–0 absorbable polylactic acid sutures.

Each rabbit received only one treatment and was considered an independent experimental unit. All 12 animals completed the study and were included in the final analysis, with no animals or samples excluded. Histological evaluation and quantitative image analysis were performed by investigators blinded to the treatment allocation.

### 2.14. Histological Evaluation

Rabbits were euthanized 4 weeks after scaffold implantation, and urethral tissues were collected for histological staining. H&E staining revealed inflammatory cell infiltration, fibroblast proliferation, angiogenesis, and re‐epithelialization at the wound site. Masson’s trichrome staining, a classic method for collagen fiber assessment, stained collagen fibers blue to evaluate collagen fiber regeneration at the wound site. Collagen synthesis is also critical for tissue repair. Staining was performed according to the product manual.

Tissues were first dewaxed and rehydrated, followed by two 15‐min treatments with xylene, two 5‐min treatments with absolute ethanol, and separate 5‐min treatments with 95% and 80% ethanol, then rinsed with water. Subsequently, staining was performed by immersing the sections in the staining solution. After staining, sections were rinsed with water. Dehydration, clearing, and mounting were followed: Sections were treated successively with ethanol solutions of varying concentrations (80%, 95%, 95%, anhydrous ethanol, and anhydrous ethanol) for 0.5 min each, followed by two rounds of xylene clearing (3 min each). Neutral resin was then used for mounting. Finally, photographs were taken under a microscope.

### 2.15. Immunohistological Evaluation

The steps of immunofluorescence experiments on paraffin sections were as follows: After tissue section dehydration, the tissue was placed in EDTA antigen retrieval buffer for antigen retrieval in a microwave oven. Then, the cells were blocked with serum, incubated with an appropriate antibody, stained with DAPI, mounted, photographed, and examined under a Nikon inverted fluorescence microscope. Semiquantitative analysis was performed using Image*J* software.

### 2.16. Data Analysis

Statistical analyses were performed using GraphPad Prism. Data are presented as mean ± standard deviation (SD). For comparisons among multiple independent treatment groups at a single time point, one‐way analysis of variance (ANOVA) followed by Tukey’s multiple‐comparison test was used. For experiments involving both treatment and time, two‐way ANOVA followed by an appropriate multiple‐comparison test was performed. Normality and homogeneity of variance were assessed before parametric analysis. For animal experiments, each rabbit was considered an independent biological replicate. Measurements obtained from multiple histological fields from the same animal were averaged to generate one value per animal before statistical analysis. A value of *p* < 0.05 was considered statistically significant.

## 3. Results

### 3.1. Characterization of BPNS

Black phosphorus (BP), a two‐dimensional inorganic material, has attracted widespread attention due to its excellent biocompatibility. As shown in Figure 1A–C, SEM and transmission electron microscopy (TEM) were used to characterize the morphology and structure of BPNS. SEM image (Figure 1A) revealed that BPNS possesses a typical two‐dimensional layered structure, with characteristic wrinkles present on the surface. TEM image (Figure 1B) demonstrated that BPNS exhibits good transmission under the electron beam, further confirming its ultrathin nature. High‐resolution transmission electron microscopy (HR‐TEM) images (Figure 1C) reveal distinct lattice fringes, with a lattice spacing estimated at 0.259 nm. This spacing corresponds to the (111) crystal plane of crystalline BP [38]. These results validate the high crystallinity and structural integrity of the BPNS.

**FIGURE 1:**
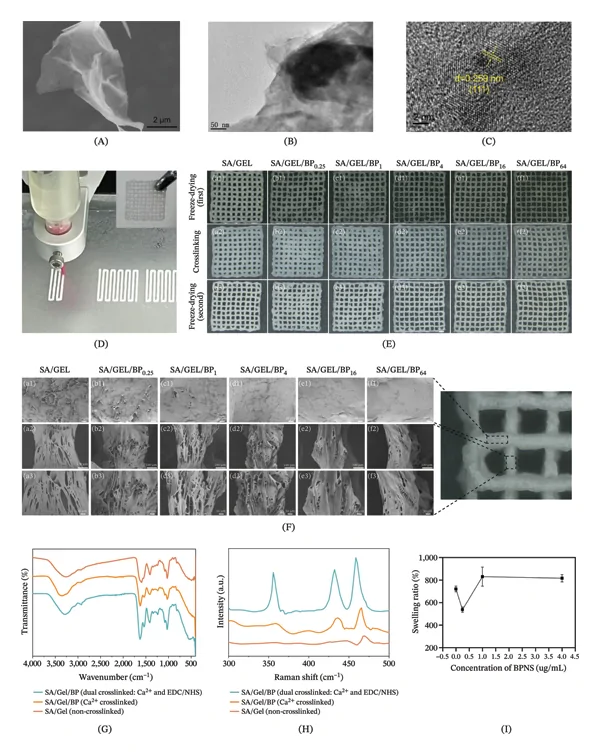
Characterization of BPNS and 3D‐printed SA/Gel/BPNS scaffolds. (A) SEM image and (B) TEM image of the commercial BPNS, showing a typical 2D nanosheet morphology. (C) HR‐TEM image of BPNS. (D) Image of the extrusion‐based 3D printing process. (E) Macromorphology of 3D‐printed scaffolds with varying BPNS concentrations (0, 0.25, 1, 4, 16, and 64 μg·mL^−1^) at different stages: first freeze‐drying, crosslinking, and second freeze‐drying. (F) Representative SEM images of SA/Gel/BPNS scaffolds containing different BPNS concentrations (0, 0.25, 1, 4, 16, and 64 μg·mL^−1^) at different magnifications, showing the interconnected porous microstructures of the corresponding scaffold formulations. (G) FTIR spectra and (H) Raman spectra of the scaffolds. (I) Swelling ratio of the composite scaffolds as a function of BPNS concentration in PBS (*n* = 3).

### 3.2. Fabrication and Characterization of SA/Gel/BPNS Hydrogels

Highly ordered grid‐like scaffolds were fabricated via an extrusion‐based 3D printing technique by optimizing the printing pressure (0.2–0.4 MPa) and scanning speed. The printability of the printable hydrogel ink was evaluated based on its ability to form continuous and uniform cylindrical filaments during the extrusion process (Figure 1D). The SA/Gel/BPNS hydrogel ink exhibited good flow properties at 37°C and was loaded into a printing syringe. Before printing, the syringe containing the ink was precooled at 4°C for 8–10 min. Meanwhile, the printing platform chamber temperature was set to 19°C. The SA/Gel/BPNS scaffold was constructed on the printing platform via layer‐by‐layer deposition.

All scaffolds underwent a “freeze‐drying–crosslinking–refreeze‐drying” process to improve structural integrity (Figure 1E). The initial freeze‐drying step generated a porous framework that helped preserve the printed grid architecture during subsequent processing and facilitated the penetration of the crosslinking agents into the scaffold. During the sequential crosslinking process, Ca^2+^‐mediated ionic crosslinking was first employed to stabilize the alginate network, followed by EDC/NHS treatment to promote covalent coupling between accessible carboxyl groups within the hydrogel network and the primary amino groups of gelatin. Previous studies have demonstrated that EDC/NHS‐mediated carbodiimide coupling promotes amide‐bond formation between carboxyl and amino groups and can improve the crosslinking stability of gelatin‐containing hydrogel networks [39]. Similar chemical crosslinking strategies have also been applied to alginate/gelatin‐based composite hydrogels to enhance network stability [40]. In the present fabrication strategy, Ca^2+^‐mediated ionic crosslinking was performed before EDC/NHS treatment to rapidly stabilize the alginate network and preserve the printed architecture during subsequent processing. Although Ca^2+^ coordination may reduce the availability of some alginate carboxyl groups for subsequent carbodiimide‐mediated coupling, EDC/NHS treatment can provide additional covalent stabilization through the remaining accessible functional groups. Therefore, the additional covalent stabilization introduced by EDC/NHS may contribute to improved retention of gelatin within the present SA/Gel network and reduce its potential diffusion or leaching compared with physical entrapment alone. Finally, secondary freeze‐drying was performed to obtain a stable and interconnected porous scaffold. Such an interconnected structure provides a favorable microenvironment for cell adhesion and growth, which is important for urethral tissue repair [41].

As shown in Figure 1E, as the BPNS concentration increased from 0 μg·mL^−1^ to 64 μg·mL^−1^, the color of the scaffold gradually changed from white to dark gray, indicating the uniform distribution of BPNS within the hydrogel matrix. SEM image (Figure 1F) reveals a highly interconnected, porous honeycomb‐like structure. Pore sizes range from 150 to 250 μm, with thin and uniform pore walls. Upon the incorporation of BPNS, the surface of the pore walls shifted from smooth to rough, with high‐magnification images revealing tiny BPNS fragments embedded in or adsorbed onto the polymer matrix. This interconnected microporous structure not only mimics the natural ECM but also provides sufficient space for fibroblast adhesion and three‐dimensional growth. The composite hydrogel ink exhibited good extrusion behavior during printing, producing continuous filaments with relatively uniform diameters and well‐defined edges. The multilayer scaffolds exhibit a connected pore structure at the microscopic level, and their printing accuracy closely matches the predesigned CAD model, demonstrating that this composite system possesses excellent printability and structural fidelity.

To elucidate the crosslinking mechanism of the composite hydrogel, FTIR and Raman spectroscopy were employed for characterization. The FTIR spectrum (Figure 1G) shows a broad band near 3300 cm^−1^ corresponding to the stretching vibrations of ‐OH and ‐NH groups. In the SA/Gel/BPNS hydrogels, slight changes were observed in the characteristic carboxyl‐related bands of SA at approximately 1610 cm^−1^ and 1415 cm^−1^, together with changes in the amide I band of Gel at approximately 1650 cm^−1^. These spectral changes suggest alterations in the local chemical environment of the corresponding functional groups within the composite hydrogel network, which may arise from the combined contributions of Ca^2+^‐mediated ionic crosslinking, EDC/NHS‐mediated covalent coupling, and intermolecular interactions among SA, Gel, and BPNS. Raman spectroscopy was used to characterize the crystal structure stability of the BPNSs (Figure 1H). Three characteristic scattering peaks were observed at 362.4 cm^−1^, 439.1 cm^−1^, and 466.8 cm^−1^, corresponding to the three vibrational modes of BP, respectively. The BPNSs in the composite hydrogel retained these characteristic peaks without significant shifts in peak positions, proving that the 3D printing and physical crosslinking processes did not induce severe oxidation or phase transitions in Bp, thereby ensuring its optoelectronic and biological activity.

Additionally, the gel’s swelling properties were evaluated, as this is a key factor in maintaining the urethral microenvironment (Figure 1I). The scaffold exhibited a significant swelling ratio of 500%–800%. The swelling capacity showed slight variations with increasing BPNS concentration. This suggests that the BPNS, acting as physical fillers, may modulate the crosslinking density and hydrophilicity of the hydrogel network. This moderate swelling capacity not only provides physical support through volume expansion but also offers an ideal moist environment for cellular activity during the repair of urethral strictures. The degradation behavior of the hydrogels was further evaluated over 30 days. As shown in Figure S1, all hydrogel groups exhibited a gradual decrease in residual mass with increasing incubation time. A relatively rapid mass loss was observed during the first 7 days, with approximately 65%–76% of the initial mass remaining. Thereafter, degradation proceeded more gradually, and approximately 50%–59% of the initial mass remained after 30 days. Similar degradation profiles were observed among the different formulations, suggesting that BPNS incorporation had only a limited influence on the overall degradation behavior of the SA/Gel hydrogel matrix.

### 3.3. In Vitro Biocompatibility and Migration of SA/Gel/BPNS Hydrogels

Throughout the entire urethral reconstruction process, it is necessary to effectively promote wound healing in the early stages and prevent scar hyperplasia in the later stages [42]. The effect of BPNS gel scaffolds at different concentrations on the migration capacity of HFFs was evaluated using a scratch assay (24 h) (Figure 2A–B). Specifically, HFFs in all groups exhibited progressive migration toward the center of the scratch, without evident cell death. Compared with the control group, the cell migration rate in the BP_0.25_ group was significantly higher, while the migration rates in the other three groups were all below 50%, indicating that low concentrations of BPNS promoted the proliferation and migration of HFFs. At the same time, as the BPNS concentration increased further, the migration rate showed a marked decrease. However, given that good biocompatibility (noncytotoxicity) is the foundation for the clinical application of materials [43], we evaluated the effects of the SA/Gel/BPNS hydrogel on cell viability at different time points (1, 3, and 5 days; Figure 2C–E). On Day 1 (Figure 2C), the OD values were comparable across all groups, indicating no significant cytotoxicity. Starting from Day 3 (Figure 2D–E), distinct differences in OD values emerged among the groups. The high‐concentration groups (16 and 64 μg·mL^−1^) exhibited significant cytotoxicity, whereas the low‐concentration groups (0.25 and 1 μg·mL^−1^) demonstrated good biocompatibility and, to some extent, promoted the sustained proliferation of HFFs. The live/dead staining results of fibroblasts seeded on the scaffold surface on Days 1, 3, and 5 are shown in Figure 2F, where live cells appear as green fluorescence and dead cells as red fluorescence. Enlarged individual images corresponding to Figure 2F are provided in Figure S2 in the Supporting Information for clearer visualization. In the printed and crosslinked hydrogel system, live/dead staining results showed that the control group and the SA/Gel/BPNS_0.25_ group consisted mainly of live cells, while the number of dead cells gradually increased with rising BPNS concentration, particularly in the high‐concentration groups (16 and 64 μg·mL^−1^). This observation is highly consistent with the CCK‐8 experimental data, jointly confirming that high concentrations of BPNS (16 and 64 μg·mL^−1^) exhibit significant cytotoxicity toward HFFs.

**FIGURE 2:**
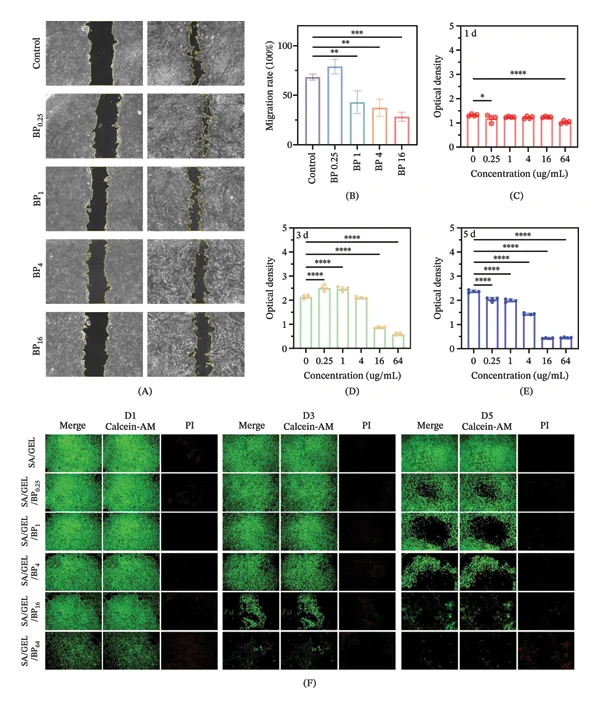
In vitro biocompatibility and migration evaluation of HFFs on SA/Gel/BPNS hydrogels. (A) Representative images of the scratch assay for HFFs at 0 and 24 h postwounding across different groups. (B) Quantitative analysis of the cell migration rate after 24 h (*n* = 3). (C–E) Cell proliferation and metabolic activity of HFFs cultured with different concentrations of BPNS (0, 0.25, 1, 4, 16, and 64 μg·mL^−1^) at Days 1, 3, and 5, as determined by CCK‐8 assay. (F) Live/dead staining of HFFs after 1, 3, and 5 days of incubation (Green: Calcein‐AM, live cells; Red: PI, dead cells). Data are expressed as mean ± SD. Statistical significance: ^∗^ *p* < 0.05, ^∗∗^ *p* < 0.01, ^∗∗∗^ *p* < 0.001, and ^∗∗∗∗^ *p* < 0.0001 compared with the control group.

### 3.4. Effects of SA/Gel/BPNS Hydrogels on Fibroblast Activation and Protein Expression

We measured mRNA expression levels in HFFs across different treatment groups, which were treated with 0.25 μg·mL^−1^ or 4 μg·mL^−1^ BPNS (Figure 3A–C). RT‐qPCR results indicated that the mRNA expression of fibronectin, COL1, and *α*‐SMA all exhibited a clear concentration‐dependent trend. Specifically, compared to the group without BPNS, the introduction of a low concentration (0.25 μg·mL^−1^) of BPNS significantly upregulated the mRNA levels of fibronectin and *α*‐SMA. When the BPNS concentration was increased to 4 μg·mL^−1^, a marked inhibitory effect was observed. Therefore, we infer that an appropriate dose of BPNS can stimulate the proliferation and phenotypic activation of HFFs, which is crucial for the tissue healing process during the early stages of urethral repair. Interestingly, the inhibitory effect observed in the BP_4_ group also suggests a biphasic regulatory effect of BPNS on fibroblast behavior.

**FIGURE 3:**
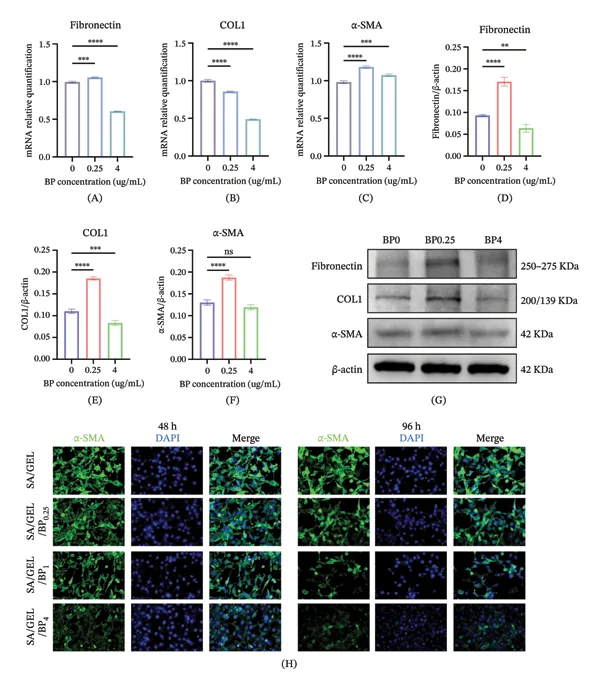
BPNS concentration‐dependent biphasic regulation of fibrotic marker expression in HFFs. (A–C) Relative mRNA expression of fibronectin, COL1, and *α*‐SMA determined by RT‐qPCR. (D–F) Quantitative analysis of protein expression normalized to *β*‐actin. (G) Representative WB bands of fibronectin, COL1, *α*‐SMA, and *β*‐actin. (H) Immunofluorescence images of *α*‐SMA (green) and DAPI (blue) in HFFs cultured with different patches at 48 h and 96 h. Data are presented as mean ± SD (*n* = 3). ^∗^ *p* < 0.05, ^∗∗^ *p* < 0.01, ^∗∗∗^ *p* < 0.001, ^∗∗∗∗^ *p* < 0.0001; ns, no significance.

WB analysis was conducted to further assess several key fibrosis‐related markers. The results of protein‐level analysis were consistent with the mRNA data (Figure 3D–G), demonstrating a significant biphasic regulatory effect. In the BP_0.25_ group, the protein expression of fibronectin, COL1, and *α*‐SMA was significantly upregulated, indicating a significant pro‐repair capacity. As a key component of the ECM, fibronectin effectively promotes cell migration, adhesion, proliferation, hemostasis, and tissue healing [44]. The accumulation of COL1 stimulates collagen deposition, suppresses excessive inflammatory responses, maintains tension around the wound, and accelerates wound healing [45]. Therefore, the upregulation of protein expression in the BP_0.25_ group indicates that BPNS at an appropriate concentration can promote the activation of HFFs and the synthesis of the ECM, thereby providing a favorable microenvironment for the early repair of urethral tissue. In contrast, under high‐concentration BPNS treatment (BP_4_), the protein expression levels of fibronectin and COL1 were significantly lower than those in the control group. This result suggests its potential to inhibit excessive fibrosis and scar formation.

Immunofluorescence staining was used to further examine changes in fibroblast (Figure 3H). At 48 h and 96 h, the BP_0.25_ group exhibited stronger *α*‐SMA fluorescence signals and more pronounced cytoskeletal structures, whereas the high‐concentration BPNS treatment group showed reduced fluorescence intensity and impaired cell spreading ability. The experimental results reveal the dual regulatory potential of BPNS on HFF behavior: Low‐concentration BPNS accelerates early healing by promoting ECM deposition, whereas higher‐concentration BPNS demonstrates a potential function in inhibiting scar hyperplasia by downregulating the expression of profibrotic markers. This concentration‐dependent phenotypic regulation capability makes the SA/Gel/BPNS hydrogel a promising candidate for effectively inhibiting fibrosis while ensuring repair quality through controlled local release.

### 3.5. In Vivo Evaluation of Urethral Stricture Repair in Rabbits

To evaluate the reparative effects of SA/Gel/BPNS scaffolds containing different BPNS concentrations in vivo, we established a rabbit urethral injury model. We created the urethral injury model by cutting the urethral mucosa with scissors to simulate the clinical condition of patients with urethral injuries. The three hydrogel groups, SA/Gel/BPNS_0_, SA/Gel/BPNS_0.25_, and SA/Gel/BPNS_4_, were sutured to the injury site. Our previous studies using a similar rabbit urethral injury model showed that the injured urethral tissue had undergone substantial healing by approximately 4 weeks after surgery [46]. Accordingly, we selected the 4‐week postoperative time point to assess tissue repair and remodeling in the present study. Macroscopic examination (Figure 4A) showed more irregular tissue morphology at the injury sites in the control and SA/Gel/BPNS_0_ groups, whereas the SA/Gel/BPNS_0.25_ and SA/Gel/BPNS_4_ groups exhibited relatively smoother tissue surfaces. Among the scaffold‐treated groups, the SA/Gel/BPNS_0.25_ group showed a relatively smooth and regular urethral surface. H&E staining (Figure 4B) showed a more continuous and organized epithelial layer in the SA/Gel/BPNS_0.25_ group, whereas the other groups exhibited varying degrees of structural disorganization and inflammatory cell infiltration. In contrast, the other groups showed varying degrees of structural disorganization and inflammatory cell infiltration. Masson’s staining (Figure 4C) was used to assess collagen deposition. The SA/Gel/BPNS_0.25_ group exhibited relatively uniform collagen distribution and a well‐organized structure, whereas the control group showed marked collagen overdeposition, indicating a high level of fibrosis. Interestingly, the SA/Gel/BPNS_4_ group exhibited significantly lower blue collagen staining intensity than the other groups, with a sparse distribution of fibers. To further evaluate tissue remodeling after scaffold implantation, immunofluorescence staining was performed to assess the expression and distribution of *α*‐SMA and COL1 (Figure 4D–E). Statistical analysis indicated that the fluorescence intensity of both *α*‐SMA and COL1 peaked in the SA/Gel/BPNS_0.25_ group. Although the expression levels of *α*‐SMA and COL1 were higher in the SA/Gel/BPNS_0.25_ group, this upregulation likely reflects enhanced ECM synthesis during the early stages of tissue repair rather than pathological fibrosis. This indicates that the scaffold has the potential to remodel smooth muscle and promote ECM deposition. Interestingly, the SA/Gel/BPNS_4_ group exhibited markedly different results. Specifically, the mean fluorescence intensity of *α*‐SMA in the SA/Gel/BPNS_4_ group was significantly suppressed, and COL1 expression levels were also lower. Together with the in vitro findings, these results further suggest that different BPNS concentrations are associated with distinct cellular and tissue responses. Overall, the SA/Gel/BPNS_0.25_ group showed favorable epithelial organization and tissue morphology at the 4‐week endpoint. Meanwhile, SA/Gel/BPNS_4_ demonstrates potential value in inhibiting excessive fibroblast activation and scar formation. This bidirectional regulatory property provides a new strategy for achieving scar‐free urethral repair through precise adjustment of BPNS concentration.

**FIGURE 4:**
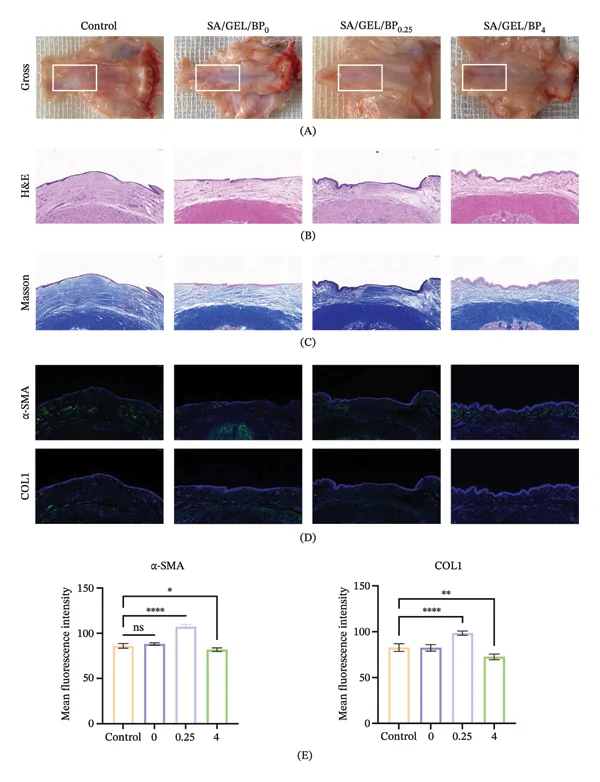
Histological and immunofluorescence evaluation of in vivo urethral repair in a rabbit model. (A) Representative gross images of the regenerated urethral tissues collected from four experimental groups: control, SA/Gel/BPNS_0_, SA/Gel/BPNS_0.25_, and SA/Gel/BPNS_4_. White boxes indicate the specific operative sites. (B) H&E and (C) Masson’s trichrome‐stained histological sections of the regenerated urethras, demonstrating the overall tissue architecture, epithelialization, and collagen deposition. (D) Immunofluorescence images displaying the spatial distribution and reorganization of key fibrotic markers within the tissue sections. Top row: *α*‐SMA (green); bottom row: COL1 (green). (E) Semiquantitative analysis of the mean fluorescence intensity for *α*‐SMA (left) and COL1 (right) from the immunofluorescence images. Data are presented as mean ± SD (*n* = 3). ^∗^ *p* < 0.05, ^∗∗^ *p* < 0.01, ^∗∗∗^ *p* < 0.001, ^∗∗∗∗^ *p* < 0.0001; ns, no significance.

### 3.6. RNA‐Seq Analysis Reveals the Molecular Mechanisms of Concentration‐Dependent Regulation

To elucidate the underlying molecular signaling pathways through which BPNS at different concentrations modulates HFF behavior, we performed transcriptomic analysis. A heatmap of DEG clusters involved in tissue repair and matrix remodeling revealed significant differences in gene expression among the experimental groups (Figure 5A). For example, in the BP_0.25_ group, genes associated with epithelial regeneration and tissue repair (such as ST14, HGFAC, and ACTA1) were significantly upregulated; whereas in the BP_4_ group, the expression of profibrotic genes (such as DPT, COL15A1, and PDGFD) was significantly suppressed. This further confirms the concentration‐dependent regulatory effect of BPNS in our 3D‐printed hydrogel patches, wherein low concentrations promote functional regeneration, while high concentrations exhibit significant antifibrotic effects. As shown in the volcano plots (Figure 5B, C), compared with the HFF control group, a total of 410 significantly upregulated genes and 212 significantly downregulated genes were identified in the BP_0.25_ group. The number of DEGs increased significantly in the BP_4_ group, comprising 3165 significantly upregulated genes and 3242 downregulated genes. The distribution of these DEGs further confirms that BPNS intervention induced a broad gene expression response. To further investigate the signaling pathways through which different concentrations of BPNS regulate HFF function, we analyzed potential biological processes using GO and KEGG enrichment analyses (Figure 5C, D). Figure 5C shows the top 15 significantly enriched GO terms in the BP_4_ group relative to the HFF group, primarily involving ECM organization, collagen fibril organization, and stress fibers. Notably, these terms closely associated with fibrosis exhibit extremely high enrichment scores, revealing the critical role of high‐concentration BPNS in inhibiting scar formation. In contrast, in the BP_0.25_ group, processes promoting healing, such as cell‐matrix adhesion and angiogenesis guidance, dominate (Figure 5C), suggesting that BP_0.25_ primarily promotes tissue regeneration by optimizing the cellular microenvironment. Through KEGG enrichment analysis (Figure 5D), we found that in the BP_4_ group, DEGs were significantly concentrated in ECM–receptor interaction, focal adhesion, and the Rap1 signaling pathway, which play central roles in mediating fibroblast activation and matrix deposition. The profound intervention of high‐concentration BPNS in these pathways provides a theoretical basis for its suppression of fibrotic scar formation. In contrast, the BP_0.25_ group exhibited a distinctly different enrichment pattern, with DEGs primarily involving various amino acid metabolic processes and the PI3K‐Akt signaling pathway. This suggests that low‐concentration BPNS focuses on optimizing the cellular metabolic microenvironment and activating pro‐regenerative signals, thereby accelerating the structural remodeling of urethral tissue. The results of the KEGG enrichment analysis once again confirm the bidirectional regulatory mechanism of BPNS, whereby it achieves both “anti‐fibrotic” and “pro‐regenerative” effects through distinct signaling branches.

FIGURE 5Transcriptomic profiling and functional enrichment analysis of HFFs treated with different concentrations of BPNS. (A) Hierarchical clustering heatmap showing the differentially expressed genes (DEGs) involved in tissue remodeling and fibrosis among the control (HFFs), BP_0.25_, and BP_4_ groups. Red: upregulation; blue: downregulation. (B) Volcano plots illustrating the distribution of DEGs in BP_0.25_ vs. HFFs (left) and BP_4_ vs. HFFs (right). Red and blue dots represent significantly upregulated and downregulated genes, respectively. (C) Gene Ontology (GO) enrichment analysis of DEGs in the BP_0.25_ group (left) and BP_4_ group (right). (D) Kyoto Encyclopedia of Genes and Genomes (KEGG) pathway enrichment analysis identifying the primary signaling pathways modulated by BPNS.

**Figure:**
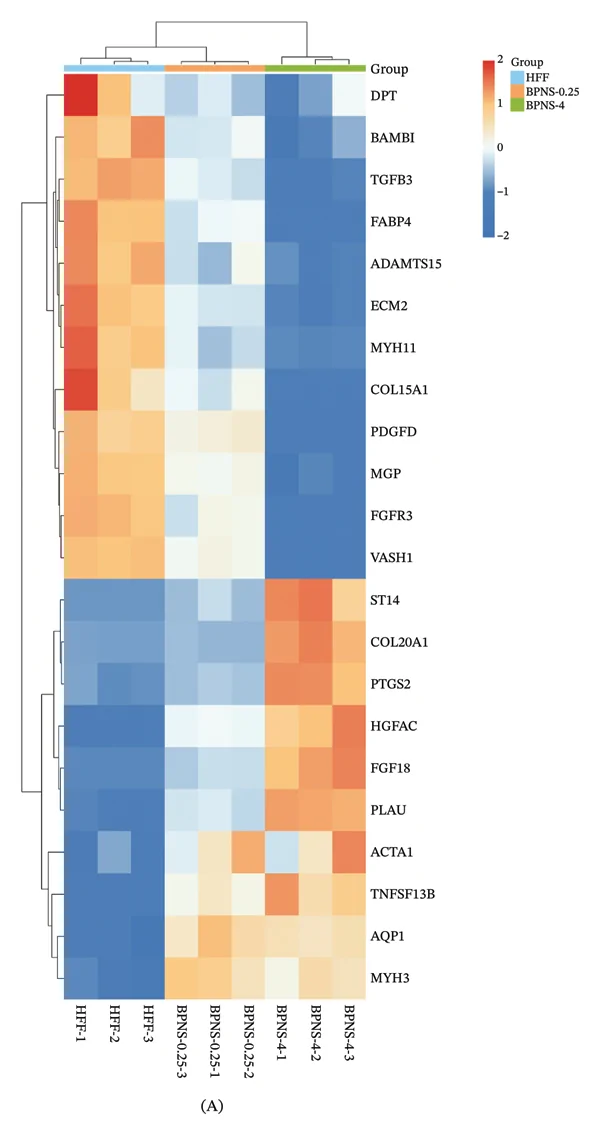

**Figure:**
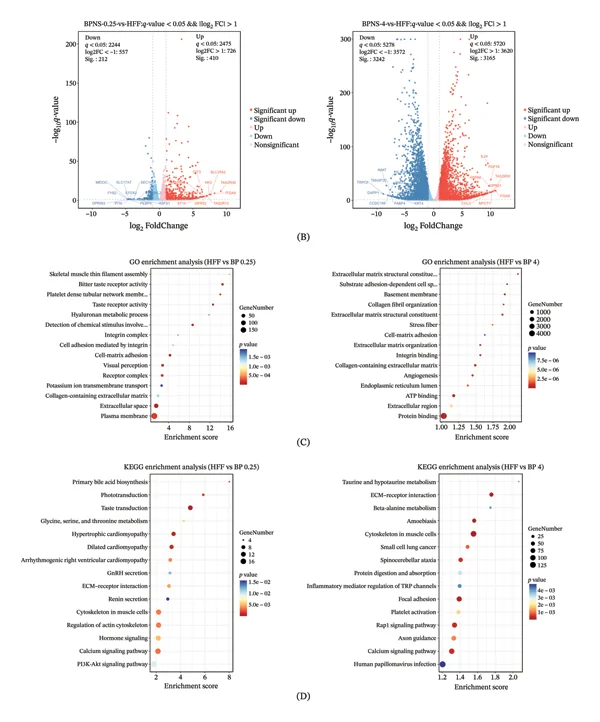

## 4. Discussion

The development of hydrogel inks with good biocompatibility and printability is crucial for extrusion‐based 3D printing [47]. Based on our previous research [43], we have chosen SA and Gel, which possess good biocompatibility and structural stability, as the base matrices of the patches. Under the printing conditions used in this study, the hydrogel ink could be continuously deposited layer by layer, and the resulting five‐layer grid construct maintained its predefined architecture after printing and subsequent processing, indicating sufficient printability and shape fidelity for fabrication of the hydrogel patches used in this study. The scaffold geometry was specifically designed as a thin patch for local urethral repair. Further optimization of printing parameters and scaffold architecture may enable the fabrication of larger and more complex free‐standing three‐dimensional constructs, thereby broadening the potential applications of this material system in urethral tissue engineering. SEM further showed that the scaffold possessed an interconnected porous structure, which could provide space for cell attachment and facilitate mass transport within the scaffold. The incorporation of BPNS into the SA/Gel matrix significantly enhanced the structural and functional properties of the hydrogel. Through in vitro and in vivo experiments, we found that BPNS exhibits a pronounced concentration‐dependent effect on fibroblast behavior. Low concentrations of BPNS (e.g., BP_0.25_) significantly promoted cell migration and proliferation, while at high doses (e.g., BP_4_), it inhibited fibroblast proliferation. At the lower BPNS concentration, the enhanced wound closure observed in the scratch assay was accompanied by transcriptomic enrichment of the PI3K‐Akt signaling pathway. PI3K‐Akt signaling is widely involved in cell survival, migration, adhesion, and tissue repair responses, and previous studies have demonstrated its important role in regulating fibroblast behavior during wound healing [48–50]. Therefore, the enrichment of PI3K‐Akt‐related signaling observed in the BP_0.25_ group may be associated with the enhanced migratory and reparative phenotype of HFFs. This interpretation is also consistent with the increased expression of fibronectin and other repair‐associated markers observed at this concentration. In contrast, the BP_4_ group showed reduced expression of fibronectin, COL1, and *α*‐SMA and exhibited a markedly different transcriptomic profile. DEGs were enriched in ECM–receptor interaction, focal adhesion, and Rap1 signaling pathways. These signaling networks are closely involved in cell–ECM adhesion, cytoskeletal organization, integrin‐mediated signaling, and matrix remodeling [51–53]. Thus, the coordinated changes in ECM–receptor interaction, focal adhesion, and Rap1 signaling may be associated with the altered fibroblast activation and matrix remodeling phenotype observed following BP_4_ treatment. This biphasic regulatory effect of BPNS on HFFs suggests that it not only constructs a bioactive microenvironment conducive to tissue regeneration but also plays a key role in suppressing pathological fibrosis. Therefore, determining the optimal concentration is crucial for maximizing its therapeutic efficacy. Our experimental results indicate that SA/Gel/BPNS hydrogel patches, characterized by a defined lattice architecture and optimized BPNS concentrations, represent a promising therapeutic strategy for pathological scarring and recurrent strictures following urethral reconstruction.

The present study demonstrates the potential of 3D‐printed SA/Gel/BPNS patches for urethral repair and provides initial insight into the concentration‐dependent biological effects of BPNS. The transcriptomic results suggest the involvement of distinct signaling pathways at different BPNS concentrations, which will require further protein‐level and pathway‐specific validation. In addition, the biological basis of the reduced cell viability observed at higher BPNS concentrations warrants further investigation. Future studies incorporating BPNS release kinetics, multiple postoperative time points, and longer‐term follow‐up, together with functional assessments, such as retrograde urethrography, urethroscopy, urethral lumen measurements, and urinary‐flow evaluation, will provide a more comprehensive understanding of BPNS exposure, tissue remodeling, urethral patency, and the potential recurrence of narrowing after scaffold implantation. The optical properties of BPNS may also offer opportunities for further functional development, including near‐infrared‐based therapy and localized drug delivery. These efforts will provide more targeted solutions for the clinical management of complex urethral strictures.

## 5. Conclusion

In this study, we used extrusion‐based 3D printing technology to fabricate a porous SA/Gel/BPNS composite hydrogel patch. In vivo studies confirmed that the patch promotes epithelial remodeling and tissue regeneration while reducing scar tissue deposition. In vitro results indicated that BPNS exerts a significant concentration‐dependent regulatory effect on fibroblast behavior. This multifunctional hydrogel system combines structural support with biological regulatory functions. Future research will further elucidate the underlying molecular mechanisms and evaluate the system’s potential for clinical translation through long‐term in vivo studies. To sum up, this 3D‐printed patch offers a highly promising bioengineering solution for the clinical treatment of urethral strictures.

## Author Contributions

Zhidong Ma: writing–original draft, methodology, investigation, and conceptualization. Jiafu Liu: methodology, investigation, and conceptualization. Wenzhuo Fang: methodology and investigation. Jie Yan: methodology and investigation. Wang Song: methodology and investigation. Xi Yang: supervision, resources, project administration, and conceptualization. Wenyao Li: review and editing, supervision, methodology, and conceptualization. Kaile Zhang: review and editing, supervision, resources, project administration, methodology, and conceptualization.

## Funding

This work was supported by the National Scientific Foundation of China (Grant Nos. 82470714, 82370684, and 82170694), the Interdisciplinary Program of Shanghai Jiao Tong University (Grant No. YG2022ZD020), the 2022 Shanghai Leading Talent Training Program (Grant No. 026), the Natural Science Foundation of Shanghai (Grant Nos. 20ZR1442100 and 22Y11905000), State Key Laboratory for Modification of Chemical Fibers and Polymer Materials (Grant No. KF2506), and the Collaborative Research Initiative on Key Diseases of Shanghai Xuhui District Health Commission (XHLHGG202109).

## Ethics Statement

All animal procedures were approved by the Institutional Review Committee of Shanghai Sixth People’s Hospital (FMX052508035).

## Conflicts of Interest

The authors declare no conflicts of interest.

## Supporting Information

Additional supporting information can be found online in the Supporting Information section.

## Supporting information


**Supporting Information** Table S1. Preparation parameters and BPNS doses of the SA/Gel/BPNS hydrogel formulations used in this study. Table S2. Primer sequences used for RT‐qPCR. Figure S1. In vitro degradation behavior of SA/Gel/BPNS hydrogels. Residual mass percentages of SA/Gel/BPNS hydrogels with different BPNS concentrations after incubation for 7, 14, 21, and 30 days. Data are presented as mean ± SD (*n* = 3). Figure S2. Live/Dead staining of HFFs after 1, 3, and 5 days of incubation (Green: Calcein AM, live cells; Red: PI, dead cells).

## Data Availability

All raw data and code are available upon request.
