# Supplementary material for: 3D‐Printed Sodium Alginate/Gelatin/Black Phosphorus Nanosheet Hydrogel Patches for Urethral Stricture Repair
Source: Int J Biomater. 2026 Oct 4;2026:5745826. doi: 10.1155/ijbm/5745826 (PMC13635782; doi:10.1155/ijbm/5745826)
Supplement: Supplementary file 1 [file IJBM-2026-5745826-s001.docx]

Supporting Information

**3D-Printed Sodium Alginate/Gelatin/Black Phosphorus Nanosheet Hydrogel Patches for Urethral Stricture Repair**

Zhidong Ma^1,#^, Jiafu Liu^1,2#^, Wenzhuo Fang^2,#^, Jie Yan^1^, Wang Song^1^, Xi Yang^3^, Wenyao Li^1,^*, Kaile Zhang^2,^*

*^1^School of Materials Science and Engineering, Shanghai University of Engineering Science, Shanghai, 201620, P. R. China*

*^2^Department of Urology, Shanghai Sixth People’s Hospital Affiliated to Shanghai Jiao Tong University School of Medicine, Shanghai, 200233, P. R. China*

*^3^Novaprint Therapeutics Suzhou Co., Ltd, Suzhou 215000, P. R. China*

*^#^Equal contribution*

***Correspondence:** Wenyao Li **(**[liwenyao@sues.edu.cn](mailto:liwenyao@sues.edu.cn)**);** Kaile Zhang **(**[great_z0313@sjtu.edu.cn](mailto:great_z0313@sjtu.edu.cn))

Table S1. Preparation parameters and BPNS doses of the SA/Gel/BPNS hydrogel formulations used in this study

| **Formulation** | **BPNS stock concentration (mg/mL)** | **Volume of BPNS stock added (μL)** | **Final precursor volume (mL)** | **Final BPNS concentration in precursor (μg/mL)** |
| --- | --- | --- | --- | --- |
| SA/Gel | 1 | 0 | 20 | 0 |
| SA/Gel/BPNS_0.25_ | 1 | 5 | 20 | 0.25 |
| SA/Gel/BPNS_1_ | 1 | 20 | 20 | 1 |
| SA/Gel/BPNS_4_ | 1 | 80 | 20 | 4 |
| SA/Gel/BPNS_16_ | 1 | 320 | 20 | 16 |
| SA/Gel/BPNS_64_ | 1 | 1280 | 20 | 64 |

Table S2. Primer sequences used for RT-qPCR.

| **Gene** | **Forward primer (5′-3′)** | **Reverse primer (5′-3′)** |
| --- | --- | --- |
| Fibronectin | GGA CCA GGT TGA TGA TAC TTC | TGT GCT ACT GCC TTC TAC TGA |
| COL1 | CCA AGA AGA CAT CCC TGA AGT | GGC AGA TAC AGA TCA AGC ATA C |
| α-SMA | CCT GAA GAG CAT CCG ACA CT | CAC AGC CTG AAT AGC CAC ATA C |
| MMP2 | CAC CAA GAA CTT CCG ATT ATC C | TGC CAT CAA AGA CAA TGT CCT |
| β-Actin | CCT CTA TGC CAA CAC AGT | AGC CAC CAA TCC ACA CAG |

**Figure S1.** In vitro degradation behavior of SA/Gel/BPNS hydrogels. Residual mass percentages of SA/Gel/BPNS hydrogels with different BPNS concentrations after incubation for 7, 14, 21, and 30 days. Data are presented as mean ± SD (n = 3).

**Figure S2.** Live/Dead staining of HFFs after 1, 3, and 5 days of incubation (Green: Calcein AM, live cells; Red: PI, dead cells).
